# Functionalising silk hydrogels with hetero- and homotypic nanoparticles

**DOI:** 10.1039/d3ra07634b

**Published:** 2024-01-22

**Authors:** Jirada Kaewchuchuen, Saphia A. L. Matthew, Suttinee Phuagkhaopong, Luis M. Bimbo, F. Philipp Seib

**Affiliations:** a Strathclyde Institute of Pharmacy and Biomedical Sciences, University of Strathclyde 161 Cathedral Street Glasgow G4 0RE UK; b Department of Pharmacology, Faculty of Medicine, Chulalongkorn University Bangkok Thailand; c Department of Pharmaceutical Technology, Faculty of Pharmacy, University of Coimbra 3000-548 Coimbra Portugal; d CNC – Center for Neuroscience and Cell Biology, Rua Larga, University of Coimbra 3004-504 Coimbra Portugal; e CIBB – Center for Innovative Biomedicine and Biotechnology, Rua Larga, University of Coimbra 3004-504 Coimbra Portugal; f Fraunhofer Institute for Molecular Biology & Applied Ecology Branch Bioresources, Ohlebergsweg 12 35392 Giessen Germany; g Friedrich Schiller University Jena, Institute of Pharmacy Lessingstr. 8 07743 Jena Germany philipp.seib@uni-jena.de +49 3641 9 499 00

## Abstract

Despite many reports detailing silk hydrogels, the development of composite silk hydrogels with homotypic and heterotypic silk nanoparticles and their impact on material mechanics and biology have remained largely unexplored. We hypothesise that the inclusion of nanoparticles into silk-based hydrogels enables the formation of homotropic and heterotropic material assemblies. The aim was to explore how well these systems allow tuning of mechanics and cell adhesion to ultimately control the cell–material interface. We utilised nonporous silica nanoparticles as a standard reference and compared them to nanoparticles derived from *Bombyx mori* silk and *Antheraea mylitta* (tasar) silk (approximately 100–150 nm in size). Initially, physically cross-linked *B. mori* silk hydrogels were prepared containing silica, *B. mori* silk nanoparticles, or tasar silk nanoparticles at concentrations of either 0.05% or 0.5% (w/v). The initial modulus (stiffness) of these nanoparticle-functionalised silk hydrogels was similar. Stress relaxation was substantially faster for nanoparticle-modified silk hydrogels than for unmodified control hydrogels. Increasing the concentrations of *B. mori* silk and silica nanoparticles slowed stress relaxation, while the opposite trend was observed for hydrogels modified with tasar nanoparticles. Cell attachment was similar for all hydrogels, but proliferation during the initial 24 h was significantly improved with the nanoparticle-modified hydrogels. Overall, this study demonstrates the manufacture and utilisation of homotropic and heterotropic silk hydrogels.

## Abstract 2

Despite many reports detailing silk hydrogels, the development of composite silk hydrogels with homotypic and heterotypic silk nanoparticles and their impact on material mechanics and biology have remained largely unexplored.

## Graphical abstract

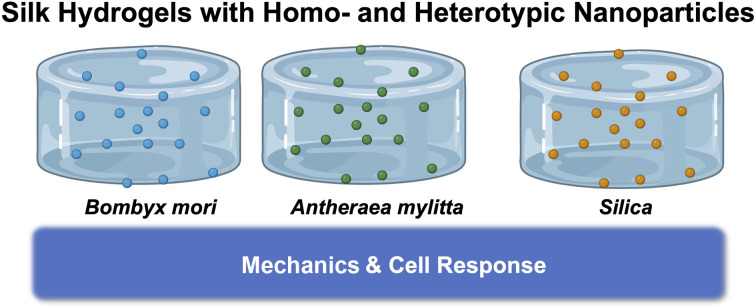

## Introduction

The tissue engineering toolbox traditionally serves to ‘repair or replace’,^1^ but it can also be deployed more widely—in disease modelling, for example.^2,3^ A key factor in tissue engineering is the cell microenvironment, as this ultimately contributes to function.^4^ This microenvironment potentially includes many different cell types and biomaterials that serve as tissue scaffolds.^5^ These scaffolds not only provide structural support but can also regulate function, including cell–cell communication, tissue homeostasis and immune cell responses (reviewed in ref. 5 and 6). Therefore, biomaterials are critical enablers of a broad spectrum of tissue engineering applications in both health and disease.

A fundamental requirement for a biomaterial is that it supports the desired function, such as cellular organisation. To date, many different biomaterials have been explored,^7^ including synthetic polymers (*e.g.* polylactide^8^ and polyethylene glycol^9^), natural polymers (*e.g.* chitosan,^10^ collagen^11^ and alginate^12^) and cell-derived extracellular matrix.^13–15^ However, all of these polymers have drawbacks, including rapid degradation, shrinkage (*e.g.* collagen), low mechanical strength (*e.g.* collagen, alginate), complicated processing (*e.g.* polyethylene glycol, alginate, extracellular matrix), biocompatibility concerns (*e.g.* leaching of chemical crosslinkers) and perturbation of the microenvironment (*e.g.* polylactide degradation causes acidification). Silk fibroin is emerging as an interesting material that shows promise for overcoming these particular limitations.^16^ Chemical modification of silk further opens up new properties to create bespoke materials with novel functions.^17^

Silk is a natural fibrous protein spun by spiders and insects (*e.g.* Lepidoptera). Silk self-assembles into a hierarchal solid fibre during the spinning process by responding to processing parameters, including shear.^18^ The most studied silk for current biomedical applications is derived from the domesticated silkworm *Bombyx mori* because its silk is clinically approved for use in humans and is readily available in large quantities due to silk farming (*i.e.* sericulture), thereby ensuring a robust silk supply chain.^19^ Silk's desirable trademarks include its remarkable mechanical properties, biocompatibility, biodegradability, hemocompatibility and water and oxygen permeability.^20,21^ Using all-aqueous processing, the silk fibre can be unspun using reverse engineering principles to yield a liquid regenerated silk feedstock (reviewed in^22^). This liquid silk can then be processed into a wide range of material formats, including films, monolithic blocks, fibres, particles, scaffolds and hydrogels,^23^ again using all-aqueous processing without any need for chemical crosslinkers or harsh solvents. Self-assembling silk hydrogels have an excellent biocompatibility track record, that also applies to the *in vivo* setting (*e.g.* ref. 24).

The *Bombyx mori* silk fibroin consists of a heavy chain of approximately 391 kDa and a light chain of approximately 26 kDa, which are linked by a single disulphide bond at the C-terminus.^25^ The C- and N-termini of the *Bombyx mori* silk heavy chain consist entirely of nonrepeating amino acid sequences and are believed to aid in the storage and self-assembly process. However, the mechanical properties of silk fibroin arise from the amino acid sequences of the silk-heavy chain that assemble into beta sheets. The copolymer-like arrangement of the silk-heavy chain contains two main motifs, namely, repetitive hydrophilic amino acid sequences and hydrophobic stretches, which result in a copolymer-like arrangement containing 11 hydrophilic and 12 hydrophobic blocks.^26^ The hydrophobic region is dominated by glycine–X repeats, where X is alanine, serine, or tyrosine. *B. mori* silk fibroin lacks the tripeptide sequence arginine, glycine, and glutamic acid (RGD) that is typically exploited by cells to mediate cell–substrate attachment *via* integrin engagement. Instead, the N terminal of the silk-heavy chain contains a fibroblast growth-promoting peptide.^27^

Lepidoptera silks share several structural features, including light and heavy chains, with conservation of the positions and spacing of cysteine residues that covalently crosslink the light and heavy chains. These silks also have an amphiphilic structure. Nevertheless, sequence specificity exists between different silkworm silks; for example, the Indian non-mulberry tasar silkworm (*Antheraea mylitta*) contains RGD sequences that are absent in *B. mori* silk, while the silk from *Antheraea assama* (golden silk) has substantial polyalanine stretches that are associated with better mechanics and thermal stability than is observed with *B. mori* silk.^28^ Therefore, possibilities exist for the creation of new materials with desired properties by blending different liquid silk types. For example, blending *Antheraea assama* with *B. mori* liquid silks triggered the solution–gel transition within 40 min of mixing. These hydrogels were physically crosslinked *via* their beta sheets and showed promising *in vivo* wound-healing properties.^19^

The present study exploits silk self-assembly using sonication as a solution–gel transition trigger.^16^ This assembly strategy has been widely used in the past because the process is simple and eliminates the need for solvents or chemical crosslinkers. Examples where these silk hydrogels have been used include soft^29^ and hard^30^ tissue engineering (*e.g.* tissue fillers, bone engineering). For example, silk hydrogels modified with several nanoparticle types, such as triphasic ceramic (Mg_2_SIO_4_, Si_3_Sr_5_, and MgO),^31^ silica,^32^ iron, silver and gold nanoparticles,^33,34^ have shown promise for a spectrum of applications, including magnetic field actuation,^35^ antibacterial functions^34^ and bone tissue engineering.^31,32^ Physically crosslinked silk hydrogels reinforced with native-like *B. mori* fibres^36^ and chemically crosslinked silk hydrogels doped with amorphous silk fibroin nanofibers^37^ have also been reported, demonstrating the possibility of tuning the silk secondary structure and fibre format. Unmodified^38^ and nanoparticle-modified silk hydrogels^39^ have also been explored for drug delivery. For example, Keiji Numata and co-workers developed the first-generation *B. mori* drug release system that incorporated silk nanoparticles within physically crosslinked silk hydrogels,^39^ while others have advanced this concept further and used these systems to release multiple drugs *in vivo*.^40^ An emerging research avenue is to use silk hydrogels as an *in vitro* tissue model that includes the tumour microenvironment.^41^

Previous studies have used silk fibroin hydrogels to assess their baseline performance as a tumour microenvironment, including the capacity to support cell migration.^42,43^ The ultimate goal of these studies is to recapitulate specific biological processes and behaviours that are dictated by the material design.^44^ In the present study we have used the human prostate cancer cell line DU145. These cells are not hormone sensitive and are moderately metastatic making them an ideal starting point for developing *in vitro* tumour models. Important factors to consider when designing these living tissue systems are the elastic and viscoelastic moduli of the extracellular mimetic matrix.^45^ For example, during solid tumour progression, the mechanical properties of the extracellular matrix change (thereby assisting disease diagnosis of solid tumours, for example). In the context of solid tumours, the substrate stiffness is accompanied by changes in flow characteristics (*i.e.* stress relaxation). Therefore, when developing extracellular matrix models, hydrogel performance is often assessed against cell function (*e.g.* cell migration, differentiation, proliferation *etc.*). Physically crosslinked silk hydrogels can mimic the three-dimensional structure of native extracellular matrix.^16^ For example, self-assembled silk hydrogels with a solid silk content of 4% w/v show viscoelasticity, which in turn impacts the cell biology.^46^

Despite many reports detailing silk hydrogels, the development of composite silk hydrogels containing homotypic and heterotypic silk nanoparticles and their impact on material mechanics and biology, has remained largely unexplored. A caveat when working with *B. mori* hydrogels is the lack of arginine–glycine–aspartic acid (RGD) sequences in this silk, as these sequences are necessary for integrin-mediated cell adhesion.^21^ For this reason, silks from non-mulberry tasar silkworms (*e.g.*, *Antheraea mylitta*) are more promising because they contain the RGD sequence. However, how well these silks allow tuning of the mechanics and cell adhesion that ultimately control the cell–material interface is unknown. Therefore, the aim of this research was to create hydrogels functionalised with nanoparticles derived from blends of *B. mori* and tasar silk to probe cell responses and to compare them to hydrogels prepared using silica nanoparticles as a reference. This work reports the manufacture of nonporous Stober silica, *B. mori* and tasar silk nanoparticles and their addition at low (0.05% w/v) and high (0.5% w/v) concentrations to 3% w/v *B. mori* silk undergoing solution–gel transition. The resulting nanoparticle-functionalised silk hydrogels had similar stiffnesses but exhibited substantial differences in stress relaxation when compared to unmodified control hydrogels. Cell attachment was similar for all the tested hydrogels.

## Materials and methods

### Silk extraction

The silk fibroin solution was prepared as detailed previously.^47^ Briefly, *B. mori* cocoons were cut into 5 mm × 5 mm pieces and degummed using 0.02 M sodium carbonate solution at 100 °C for 60 min. The degummed silk fibres were cooled to room temperature, rinsed three times with deionised water and dried in a fume hood overnight. The dried silk was dissolved in fresh 9.6 M LiBr solution at 60 °C for 3 h. This solution was dialysed against deionised water using a dialysis cassette (molecular weight cut-off 3500 Da) and the water was changed at 1, 3 and 6 h on the first day, on the next morning and evening, and again on the following morning. The silk solution was collected and centrifuged twice for 20 min at 5 °C and 9500 × *g*. The resulting silk solution was stored at 4 °C until use.

*Antheraea mylitta* silkworm cocoons were prepared based on previous work by others.^48^ Briefly, dried cocoons were cut into 5 × 5 mm pieces and 5 g samples were degummed with 2 L of 0.025 M Na_2_CO_3_ for 60 min, followed by 60 min in 2 L of 0.0125 mM Na_2_CO_3_. The silk fibres were then washed three times with 1 L of distilled water for 20 min and then dried in a fume hood overnight. The dried silk fibroin was dissolved in 1 N NaOH at a silk to NaOH ratio of 1 g to 25 mL. The samples were kept at 25 °C for up to 16 h under constant stirring at 250 rpm. Insoluble material was removed by centrifugation for 20 min at 9500 × *g*. The supernatant was transferred to a dialysis cassette (molecular weight cut-off 3500 Da; Thermo Fisher Scientific Inc., Waltham, MA, USA) and dialysed against distilled water, with four water changes over the 24 h dialysis period. The dialysed silk fibroin solution was collected and centrifuged twice at 9500 × *g* for 20 min to remove any remaining aggregates. Samples were freeze dried and reconstituted to 4% (w/v) and stored at 4 °C until use. The tasar silk fibroin concentration was calculated using the bicinchoninic acid assay protein assay and bovine serum albumin as a protein standard (Pierce™ BCA Protein Assay Kit, Thermo Fisher Scientific).

### Particle synthesis & characterisation

We used the Stober protocol to generate nonporous silica particles 100–150 nm in size.^49^ Briefly, ammonium hydroxide (NH_4_OH) served as the basic catalyst for the Stober silica reaction. Previous work showed that the concentration of NH_4_OH was directly proportional to the size of silica nanoparticles.^50^ Therefore, the volume of added 28% NH_4_OH was reduced from 3.0 mL to 1.5 mL. The NH_4_OH was added to 50.0 mL of 95% ethanol/ultrapure water and stirred for 5 min, followed by the addition of 1.5 mL tetraethyl orthosilicate (TEOS). The reaction mixture was stirred at 18 °C for 6 h and the final silica product was centrifuged at 48 384 × *g* for 15 min. The solid white product was washed three times with ethanol and dried overnight at 80 °C.

**Fig. 1:**
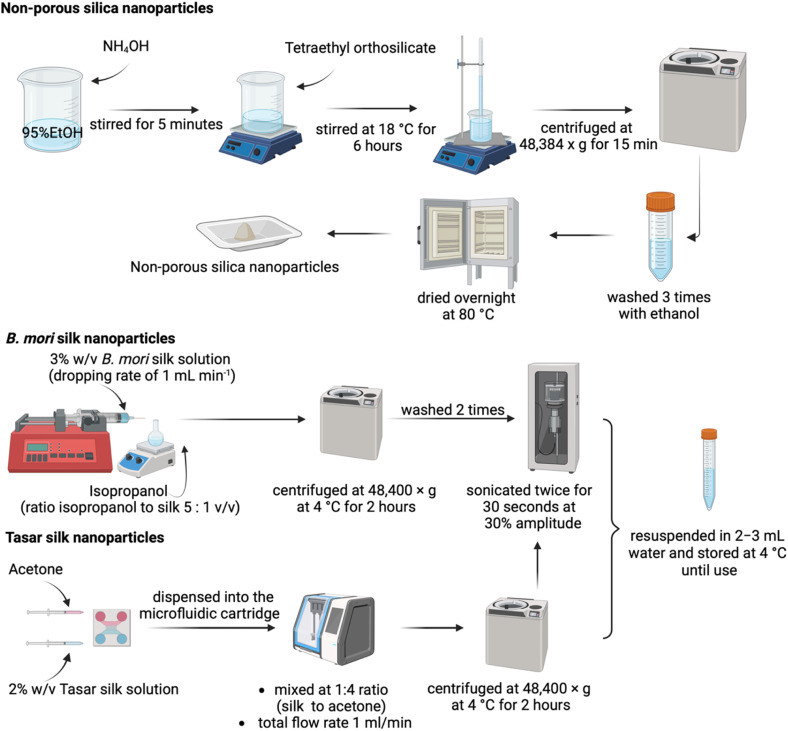
Flow diagram of nanoparticle manufacture. Silica, *B. mori* and tasar silk nanoparticle synthesis.

*B. mori* silk nanoparticles were manufactured as described previously (Matthew *et al.*, 2020). Briefly, silk nanoparticles were generated using semi-automated nanoprecipitation by controlling the silk and solvent flow with a syringe pump (Harvard Apparatus 22, Holliston, MA) (Fig. 1). The system was equipped with a syringe and blunt needle (23 G × 0.25′′) and operated at room temperature. Isopropanol was contained in a short-neck round-bottom flask and the ratio of isopropanol to silk was set at 5 : 1 (v/v). A 3% (w/v) *B. mori* silk solution was added dropwise at a rate of 1 mL min^−1^. The resulting suspension was transferred to polypropylene ultracentrifugation tubes, the volume was made up to 43 mL with distilled water and the tubes were centrifuged at 48 400 g for 2 h at 4 °C (Beckman Coulter Avanti J-E equipped with a JA-20 rotor). The supernatant was discarded and the pellet was resuspended in 20 mL distilled water and sonicated twice for 30 s at 30% amplitude with a Sonopuls HD 2070 sonicator (ultrasonic homogeniser, Bandelin, Berlin, Germany) fitted with a 23 cm long sonication tip (0.3 cm diameter tip and tapered over 8 cm). Next, 23 mL of distilled water was added and the sonicated material was centrifuged. This washing and resuspension of the pellet was repeated twice. The final pellet was collected and resuspended in 2–3 mL water. This final silk nanoparticle suspension was stored at 4 °C until use.

**Fig. 2:**
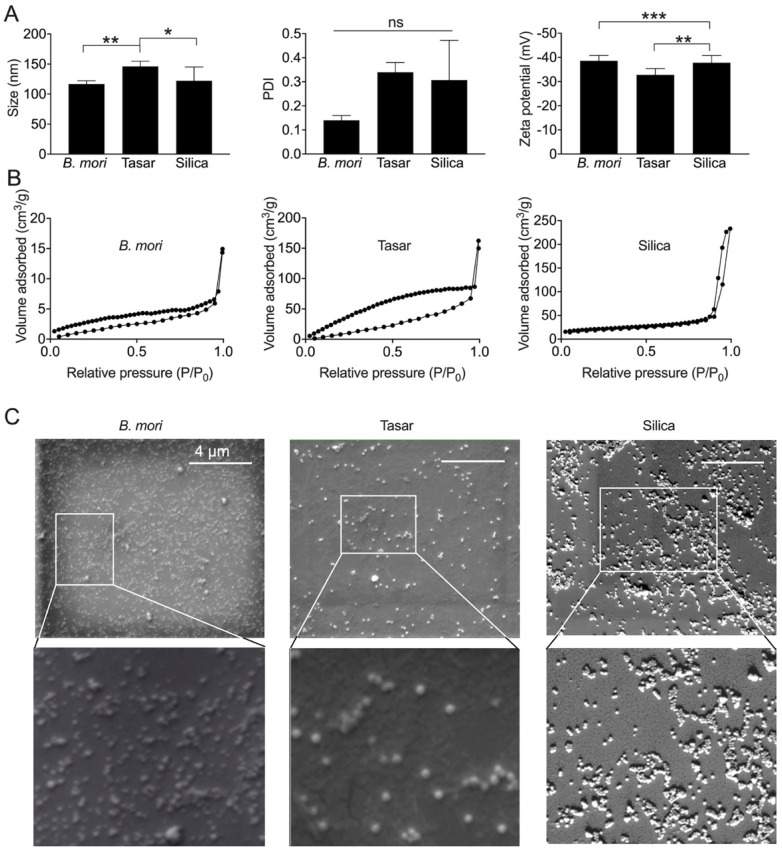
Nanoparticle characterisation of silica, *B. mori* silk, and *A. mylitta* (tasar) silk nanoparticles. (A) Particle diameter (measured by dynamic light scattering [DLS]), polydispersity (PDI) and zeta potential values. Data are presented as mean ± SD, *n* = 6 independent measurements, (B) surface area and pore volume measurements using nitrogen adsorption. *n* = 1 from 6 pooled batches, and (C) morphology of nanoparticles by scanning electron microscopy (scale bar, 4 μm; zoom, 2 μm). One-way analysis of variance (ANOVA) followed by Tukey's multiple comparison test, ns: not significant.

Tasar silk nanoparticles were synthesised using a NanoAssemblr microfluidic system (NanoAssemblr™ Benchtop Instrument version 1.5, Canada). Prefilled syringes containing 2% (w/v) silk solution and acetone were dispensed into a microfluidic cartridge and mixed at a 1 : 4 ratio (v/v) of silk solution to acetone at a total flow rate of 1 mL min^−1^ (Fig. 2). The precipitated silk nanoparticles were collected and centrifuged at 48 400 × *g* for 2 h, the supernatant was aspirated, and the pellet was resuspended in distilled water, vortexed and subsequently sonicated twice for 30 s sonication cycles at a 30% amplitude. The washing steps were repeated at least twice more. The silk particles were then stored at 4 °C until use.

### Particle characterisation

#### Size and zeta potential

The particle diameters and surface charges of silica and silk nanoparticles were determined as detailed previously.^47,51^ Briefly, silica nanoparticles were suspended in distilled water at 1 mg mL^−1^, vortexed for 10 min and sonicated for 30 s, while silk nanoparticles were vortexed for 20 s and sonicated twice at 30% amplitude for 30 s. The nanoparticle sizes were measured using a Zetasizer Nano ZS (Malvern Instruments, UK) at 25 °C. Particle sizes were determined using dynamic light scattering with a refractive index of 1.33 and 1.43 for distilled water and silica, respectively. The protein refractive index of 1.60 was used for silk nanoparticles. All measurements were performed in triplicate.

#### Pore volume and surface area of nanoparticles

The surface areas and pore sizes of the silica and silk nanoparticles were determined based on previous methodology.^52,53^ Nanoparticles were characterised by nitrogen adsorption at 77 K using a Quantachrome Autosorb IQ2 analyser (Quantachrome Instruments, Boynton Beach, Florida, USA). First, 200 mg of silica nanoparticles were degassed under vacuum at 200 °C for 18 h and 100 mg of silk nanoparticles were degassed under vacuum at 100 °C for 10 h. (All samples were outgassed at room temperature.) The samples were analysed using the ASiQwin software Model 6 version 3.0-3.01. Particle surface areas were determined using the Brunauer–Emmett–Teller (BET) method and the pore volume was determined using the Barrett, Joyner, Halenda (BJH) method.^52^

#### Scanning electron microscopy (SEM) of nanoparticles

Nanoparticle suspensions were adjusted to a final concentration of 1 mg mL^−1^, and 20 μL were pipetted onto a silicon wafer and lyophilised for 24 h at −10 °C and 0.14 mbar. The silica nanoparticles were sputter coated with 15 nm of gold using an ACE200 low-vacuum sputter-coated (Leica Microsystems, Wetzlar, Germany). Silicon wafers with nanoparticles were fixed to aluminium pin stubs with double-sided adhesive carbon tape. Specimens were imaged using the Quanta FEG-ESEM (FEI Company, Hillsboro, OR, USA; now part of Thermo Fisher Scientific Inc., Waltham, MA, USA) with a 5 kV voltage at three magnifications (5000×, 20 000×, and 40 000×). The images were processed using ImageJ for Windows 1.8.0.

### Preparation of self-assembling silk hydrogels

The preparation of self-assembling silk hydrogels was described previously.^54^ Briefly, 4 mL of 3% w/v silk solution was prepared and transferred to 15 mL Falcon tubes. The sample was then sonicated using a digitally controlled probe sonicator (Sonopuls HD 2070, Bandelin, Berlin, Germany) fitted with a 23 cm long sonication tip (0.3 cm diameter tip and tapered over 8 cm) at 30% amplitude for typically 4 sonication cycles on ice (one cycle consisted of 30 s on and 30 s off) to initiate the solution–gel transition. Silk hydrogels containing nanoparticles were generated by adding and mixing 0.05 or 0.5% w/v of silica, *B. mori* or tasar silk nanoparticles to samples prior to the completion of the solution–gel transition.

### Silk secondary conformation analysis by Fourier-transform infrared spectroscopy (FTIR)

The secondary structures of *B. mori* and tasar silk nanoparticles and silk hydrogels were measured as detailed previously (Matthew *et al.*, 2020). Briefly, silk nanoparticles and silk hydrogels were frozen overnight at −80 °C and lyophilised. The secondary silk structure was measured with a TENSOR II FTIR spectrometer (Bruker Optik GmbH, Ettlingen, Germany). The samples were scanned 32 times for background and 128 times for the samples at 4 cm^−1^ resolution over the wavenumber range of 400–4000 cm^−1^. The secondary structures were assigned as detailed previously. Baseline and peak fits were corrected using OriginPro 2021 software. The amide I region (1595–1705 cm^−1^) was identified and deconvoluted as follows: 1605–1615 cm^−1^ as side chain/aggregated strands, 1616–1637 cm^−1^ and 1697–1703 cm^−1^ as beta-sheet structure, 1638–1655 cm^−1^ as random coil structure, 1656–1662 cm^−1^ as alpha-helical bands and 1663–1696 cm^−1^ as beta-turns. Air-dried films were used as negative controls and a 70% ethanol-treated silk film was used as a positive control.

### Rheology of silk hydrogel composites

Hydrogels were manufactured as detailed above. Samples undergoing the solution–gel transition were transferred and allowed to set in casting moulds (11 mm diameter with an average thickness of 9 mm). The hydrogels were equilibrated in phosphate buffered saline (PBS) overnight and then subjected to rheology characterisation (Haake Mars Liquid Rheometer, Thermo Fisher Scientific) at 25 °C using a 20 mm diameter plate and appropriate gap size. The storage modulus (*G*′) was measured using a time sweep over a strain of 0.01–100% at a frequency of 1.0 Hz. The stress-relaxation rate (*G*′′) was measured at a 15% strain to mimic the human extracellular matrix.^55^ The stress relaxation was recorded every 10 s for a total of 300 s. Stress was normalised by the initial stress, and the half stress relaxation time, which is the time that the stress is relaxed to half of the initial stress, was calculated as detailed previously.^46^

### Cell culture

The human prostate cancer DU145 cell line was purchased from the American Type Culture Collection (Manassas, VA, USA). Briefly, the cells were routinely cultured in tissue culture–treated polystyrene flasks in Roswell Park Memorial Institute (RPMI) 1640 medium, supplemented with 10% v/v foetal bovine serum, 50 U per mL penicillin and 50 μg per mL streptomycin. Unless otherwise indicated, the cells were seeded at 5000 cells per cm^2^. The cells were cultured at 37 °C in 95% relative humidity and 5% CO_2_ and passaged using trypsin and standard protocols. For cultures using silk hydrogels, the substrates were prepared in 96-well plates as described previously.^46^

#### Quantification of cell attachment

DNA quantification was used to determine cell numbers, as detailed previously.^46^ Briefly, the Quant-iT™ PicoGreen kit was used to measure the number of DU145 cells that had attached to the control and silk hydrogel substrates at 2, 4 and 24 h post seeding. At the indicated time point, the culture medium was removed and replaced with 200 μL of PBS. The cells were incubated for 3 h in a humidified atmosphere of 5% CO_2_ at 37 °C and then homogenised and digested at 60 °C for 16 h in 200 μL papain buffer (5 mg mL^−1^ papain, 2 mM cysteine, 50 mM sodium phosphate and 2 mM ethylenediaminetetraacetic acid at pH 6.5 in nuclease-free water). The papain-digested samples were collected and centrifuged for 5 min at 13 000 × *g* to eliminate cellular debris. The supernatants were collected and dDNA was quantified with the Quant-iT™ PicoGreen kit, following the manufacturer's protocol. Blank hydrogels from the same time points were used as controls to account for background fluorescence.

### Statistical analyses

Data were plotted and analysed as detailed previously.^56^ Briefly, sample pairs were analysed by one-way ANOVA with Tukey's multiple comparison post hoc test (Prism 9.2.0; GraphPad Software Inc., San Diego, CA, USA). Asterisks were used to denote statistical significance, as follows: *P < 0.05, **P < 0.01, ***P < 0.001. All data were presented as mean values ± standard deviation (SD). The number of independent experiments (*n*) is noted in each figure legend.

## Results & discussion

Tissue engineering principles are not only relevant in health applications, but they are also important when designing *in vitro* disease models.^2,3^ One key requirement is to create a cell microenvironment that supports tissue development; therefore, biomaterials are critical enablers. One emerging material is silk, as silk fibroin is well-placed to develop disease models due to its tuneable mechanical properties and biocompatibility. Importantly, silk hydrogels do not shrink *in vitro* over time (a common problem when working with collagen).^29^ Self-assembled silk hydrogels are particularly interesting because they show viscoelastic material properties that resemble those of native extracellular matrix and their mechanics can be tuned by increasing the silk content.^46^ For example, Liu *et al.* fabricated silk hydrogels reinforced with fibroin nanofibers to enhance the mechanical properties and found that this reinforcement strategy enhanced the stiffness of the silk fibroin hydrogel (from 0.6 to 160 kPa).^37^

One limitation when working with *B. mori* silk fibroin is its lack of the RGD sequence necessary for integrin-mediated cell adhesion.^19^ For this reason, *B. mori* silk hydrogels can be viewed as ‘blank slates’ that require modification to maximise their potential. Tasar silk does contain the RGD sequence, but no examples currently exist in the literature of *B. mori* hydrogels functionalised with tasar silk nanoparticles. Therefore, the present work closes a critical knowledge gap.

The use of tasar silk nanoparticles to functionalise *B. mori* silk hydrogels is ideal because it permits spatial control of RGD functionalisation. In the present work, nonporous silica nanoparticles synthesised using the Stober method^49^ were used as a reference. The *B. mori* silk nanoparticles were manufactured using the Matthew semi-batch set-up^57^ and tasar silk nanoparticles were synthesised using microfluidic-assisted antisolvent precipitation.^48^ All three nanoparticle types were characterised according to their size, polydispersity index and zeta potential using dynamic light scattering and zeta potential measurements, as our aim was to work with particles that were of similar size and surface charge. We therefore tuned the silica particle size by adjusting the NH_4_OH concentration (data not shown), because increasing ammonium hydroxide concentration is known to increase the nanoparticle size.^50^ Ultimately, all three nanoparticle types had a size range of 120 to 150 nm, a zeta potential between −33 and 39 mV and similar polydispersity indices that were not statistically different. Specifically, *B. mori* silk nanoparticles had an average size of 117 ± 5.31 nm, a polydispersity index of 0.14 ± 0.02 and a zeta potential of −38.6 ± 2.23 mV (Fig. 2A), the *Antheraea mylitta* (tasar) silk nanoparticles had an average size of 146 ± 8.64 nm, a polydispersity index of 0.34 ± 0.04 and a zeta potential of −32.8 ± 2.64 mV, and the silica nanoparticles had an average size of 122 ± 22.68 nm, a polydispersity index of 0.31 ± 0.17 and a zeta potential of −37.8 ± 2.98 mV (Fig. 2A). These silk nanoparticle results are consistent with previous work using *B. mori*^57–59^ and tasar^48^ silk stocks.

The pore volumes and surface areas of the nanoparticles were determined using nitrogen adsorption. The single-point Brunauer–Emmett–Teller analysis showed that *B. mori* and tasar silk nanoparticles had pore volumes of 0.023 and 0.094 cm^3^ g^−1^, respectively. This pore volume determination may indicate particle aggregation^60^ because electron microscopy studies have suggested that silk nanoparticles are solid. The surface areas of the *B. mori* and tasar silk nanoparticles were determined to be 5.02 and 33.65 m^2^ g^−1^, respectively (Fig. 2B).

Our silica nanoparticles were classified by a type II isotherm, indicating that they were nonporous, as would be expected for Stober silica nanoparticles^60^ The presence of a hysteresis loop classified the material as an H1 type, indicative of nanoparticle aggregation (rather than porosity). Both the single-point Brunauer–Emmett–Teller and Barrett–Joyner–Halenda measurements revealed that the silica nanoparticles had a pore volume of 0.350 cm^3^ g^−1^ and an average surface area of 62.77 m^2^ g^−1^ (Fig. 2B). These types of values are typical for Stober silica nanoparticles, as reported in the reference literature.^60^

Overall, based on IUPAC guidelines,^61^ the silica, *B. mori* and tasar silk nanoparticles were non-porous and classified as type II materials because their isotherm graphs showed a monolayer adsorption up to high *P*/*P*_o_. We then assessed the morphology of the nanoparticles by scanning electron microscopy. Both *B. mori* and tasar silk nanoparticles had spherical shapes with sizes similar to those determined by dynamic light scattering (Fig. 2C). These results correlated well with previous reports (*e.g.* ref. 48 and 57–59). The Stober silica nanoparticles also had a spherical shape and an average size similar to that determined by dynamic light scattering, again in agreement with previous reports (*e.g.* ref. 49 and 50).

**Fig. 3:**
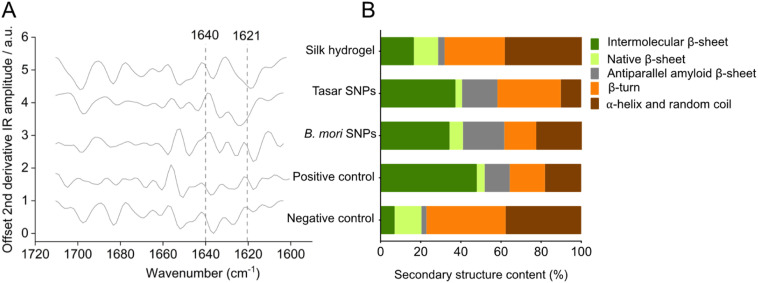
Fourier-transform infrared (FTIR) spectra and peak assignments. (A) FTIR spectra of *B. mori* and tasar silk nanoparticles, silk hydrogels, air-dried silk film (negative control), and 70% ethanol-treated silk film (positive control). (B) Analysed secondary structure.

FTIR spectroscopy was used to determine the silk secondary structure and the different functional groups present in the silica nanoparticles. The Stober silica nanoparticles showed a typical absorption peak of Si–O–Si (797.45 cm^−1^ and 1062.79 cm^−1^) and Si–OH asymmetric stretching vibration (945.81 cm^−1^) (data not shown), in agreement with previous reports.^62^ The secondary structure of the silk nanoparticles and hydrogels indicated extensive β-sheets in our self-assembled silk hydrogels and particles (Fig. 3), consistent with other literature (*e.g.* ref. 57 and 63). The FTIR results showed an amide I absorption peak at 1600–1700 cm^−1^ for all silk samples (Fig. 3A). Comparison of the spectra from *B. mori* and tasar silk nanoparticles and hydrogel composite nanoparticles to the spectra from air-dried silk films (negative control) and silk films treated with 70% v/v ethanol/distilled water (positive control) revealed the highest β-sheet content in the positive control silk films (Fig. 3B). The β-sheet contents of *B. mori* and tasar silk nanoparticles were 62% and 58%, respectively, whereas the β-sheet content of the silk hydrogels and air-dried silk films (negative control) were 32% and 23%, respectively (Fig. 3B). Overall, these data correlated well with our own studies^57^ and previous studies by others.^48,64^

We also assessed the impact of the incorporation of homo- and heterotypic nanoparticles on hydrogel mechanics. We speculated that the formation of these composites would result in different mechanics than those observed in the pristine silk hydrogels. This speculation was based on the ability of nanoparticles to orchestrate hydrogel interactions, including the formation of dynamic nanoparticle–hydrogel structures or hydrogels with adhesive surface properties. These systems typically exploit polymer–nanoparticle interactions that impact the overall bulk properties mediated by surface adsorption, such as those occurring between silica nanoparticles and polyethylene glycol (PEG),^65^ between hydrophobically modified cellulose derivatives and PEGylated polylactide nanoparticles^66^ or between TM50 silica and polyacrylamide hydrogels.^67^

**Fig. 4:**
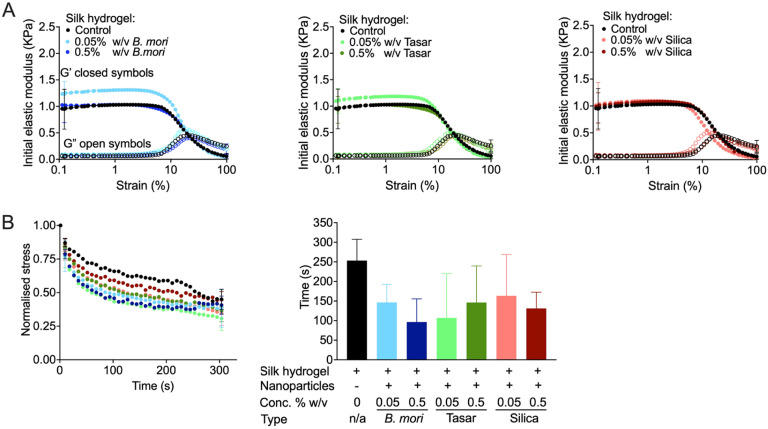
Impact of the nanoparticle concentration and type on silk hydrogel rheological properties. (A) Rheological behaviour of 3% w/v silk hydrogels doped with nanoparticles, (B) stress relaxation time and normalised stress relaxation time. Control refers to an undoped silk hydrogel. Data are presented as mean ± SD, *n* = 3 independent experiments. Closed symbols *G*′ and open symbols *G*′′.

Nanoparticle shape also affects hydrogel performance.^68^ Previous work has shown that silk reinforcement with silica increased bulk stiffness.^32^ However, studies on silk hydrogels containing nanoparticles are lacking, so little is known regarding their mechanics or cellular responses. In the present study, the flow behaviour of silk hydrogels in the presence of nanoparticles is reported by comparing the stiffness of a 3% w/v silk hydrogel with similar silk hydrogels containing *B. mori* silk, tasar silk and non-porous silica nanoparticles (Fig. 4A). However, the initial elastic moduli did not differ for any of the tested hydrogels. For example, the stiffness was 1.23 kPa for the hydrogel containing 0.05% w/v *B. mori* silk (this was the highest measured value) and 1.10 kPa for the hydrogel containing 0.05% w/v tasar silk nanoparticles. By contrast, the stress relaxation and the half stress-relaxation time showed some particle dependence, as silk hydrogels containing 0.5% w/v *B. mori* nanoparticles had the lowest value (97 s) (Fig. 4B), while pure silk hydrogel had the highest value (312 s). These values were 163 s for silk hydrogels containing 0.05% (w/v) Stober silica nanoparticles and 146 s for silk hydrogels containing 0.5% w/v *B. mori* silk nanoparticles (Fig. 4B). Overall, these trends indicated that the interactions between the nanoparticles and the silk hydrogel did not significantly alter their behaviours. Possibly, the formation of physical crosslinks between the silk molecules that are responsible for the formation of the silk hydrogel was only slightly influenced by the nanoparticle doping. Perhaps the use of sequence-coded nanoparticles will provide better control over beta-sheet bulk assembly.

**Fig. 5:**
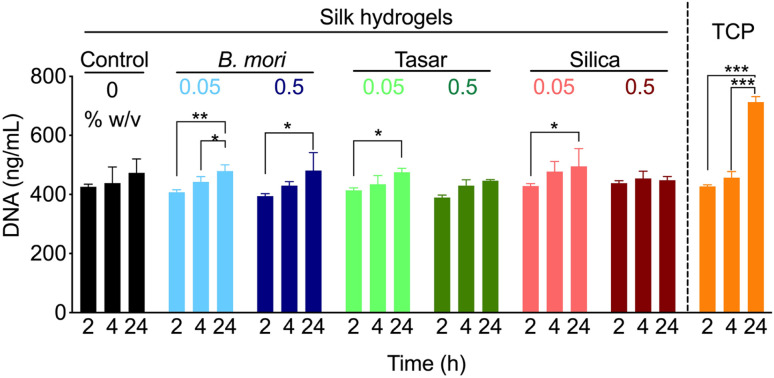
Silk hydrogels are used for cell culture studies. Monitoring DU145 cell attachment using PicoGreen kit assay at 2, 4 and 24 h. Control: silk hydrogel. TCP: tissue culture treated polystyrene. Data are presented mean ± SD, *n* = 3 independent biological experiments. One-way analysis of variance (ANOVA) followed by Tukey's multiple comparison test.

The preliminary biological responses of the nanoparticle-doped silk hydrogels were also assessed. Prior work showed that embedding silica nanoparticles within silk fibroin hydrogels enhanced the bulk mechanical properties of the hydrogel while promoting mesenchymal stem cell adhesion, proliferation, and osteogenic differentiation.^32^ In the present study, the addition of nanoparticles typically showed no significant differences for DU145 cell attachment when compared to unmodified silk hydrogels at matched timepoints (Fig. 5). However, within groups statistically significant differences were observed indicating improved cell attachment for 0.05% w/v tasar and silica nanoparticles functionalized hydrogels as well as for both 0.05 and 0.5% w/v *B. mori* nanoparticle hydrogels. However, the largest increase in DU145 cell numbers within 24 h was observed using tissue culture–treated polystyrene, which outcompeted all the silk substrates. This trend continued into days 6 and 9 of culture (data not shown). Therefore, the tasar nanoparticles used here apparently had little impact on cell attachment. This was surprising because the presence of the RGD motif in this silk had been expected to improve cell–material interaction *via* integrin engagement. This lack of improvement in cell attachment could have several reasons, including restricted accessibility of the tasar nanoparticles for integrin receptor engagement. Focal adhesion organisation shows a high sensitivity to ligand spacing, with a nanoscale average RGD spacing of 44 nm needed to form lipid raft domains at focal adhesion sites.^69^ This spacing mimics the RGD spacing found in the fibronectin.^70^ Therefore, further work is needed to improve the nanoparticle placement and spacing in our silk hydrogels. Subsequent studies can then monitor integrin engagement that will ultimately help to further characterise these substrate–cell interactions. Notably, cell viability was maintained on the silk hydrogels spiked with *B. mori*, tasar silk and silica nanoparticles throughout the culture period (Fig. 5). The present study used DU145 cells only and there is now scope to expand this work. For example, the use of prostate cancer cells that are hormone responsive or those that show a greater metastatic potential than DU145 cells^13^ would help to assess, and potentially unlock, the fully potential of these culture systems. Furthermore, the use of other cell types including reference cell lines (*e.g.* L929 fibroblasts^71^), mesenchymal stem cells^15^ or keratinocytes^72^ would further broaden the impact of this work and potentially uncover cell type specific effects. However, more work is needed to fine-tune the cell material interactions for further improvement of the biological response. We speculate that the use of sequence coded nanoparticles will enhance their interactions with the hydrogel *via* beta sheet engagement ultimately providing greater control over particle presentation. Also the use of larger particles (*e.g.* 500 nm, 1 μm and 5 μm) would enable more integrin engagement by overcoming the critical RGD spacing. The present study used 2D cultures. However, our system is readily adapted to 3D cell cultures enabling greater probing of the cell–material interface and the subsequent biological response.

## Conclusions

Overall, this study explored the effects of adding various nanoparticle types on the material mechanics and biological interactions of composite self-assembling silk hydrogels. Silica, *B. mori* silk and tasar silk nanoparticles were used to alter the stress relaxation of silk hydrogels to improve initial cell proliferation now opening up these systems to fine-tuning. The present findings demonstrated the creation and application of homo- and heterotypic silk hydrogel composites.

## Author contributions

J. K. designed, collected, analysed and interpreted the data and generated the manuscript draft. S. P. generated tasar silk nanoparticle protocol. S. A. L. M. supported the FTIR analyses. L. M. B. and F. P. S. provided training, advised on experimental design and contributed to the interpretation of the results. All authors discussed the results and/or provided advice on the experimental analysis. L. M. B. and F. P. S. supervised the project and content-edited the manuscript.

## Conflicts of interest

There are no conflicts to declare.

## Supplementary Material
